# Methodology capture: discriminating between the "best" and the rest of community practice

**DOI:** 10.1186/1471-2105-9-359

**Published:** 2008-09-01

**Authors:** James M Eales, John W Pinney, Robert D Stevens, David L Robertson

**Affiliations:** 1 Faculty of Life Sciences, University of Manchester, Manchester, UK; 2 School of Computer Science, University of Manchester, Manchester, UK

## Abstract

**Background:**

The methodologies we use both enable and help define our research. However, as experimental complexity has increased the choice of appropriate methodologies has become an increasingly difficult task. This makes it difficult to keep track of available bioinformatics software, let alone the most suitable protocols in a specific research area. To remedy this we present an approach for capturing methodology from literature in order to identify and, thus, define best practice within a field.

**Results:**

Our approach is to implement data extraction techniques on the full-text of scientific articles to obtain the set of experimental protocols used by an entire scientific discipline, molecular phylogenetics. Our methodology for identifying methodologies could in principle be applied to any scientific discipline, whether or not computer-based. We find a number of issues related to the nature of best practice, as opposed to community practice. We find that there is much heterogeneity in the use of molecular phylogenetic methods and software, some of which is related to poor specification of protocols. We also find that phylogenetic practice exhibits field-specific tendencies that have increased through time, despite the generic nature of the available software. We used the practice of highly published and widely collaborative researchers ("expert" researchers) to analyse the influence of authority on community practice. We find expert authors exhibit patterns of practice common to their field and therefore act as useful field-specific practice indicators.

**Conclusion:**

We have identified a structured community of phylogenetic researchers performing analyses that are customary in their own local community and significantly different from those in other areas. Best practice information can help to bridge such subtle differences by increasing communication of protocols to a wider audience. We propose that the practice of expert authors from the field of evolutionary biology is the closest to contemporary best practice in phylogenetic experimental design. Capturing best practice is, however, a complex task and should also acknowledge the differences between fields such as the specific context of the analysis.

## Background

As scientists, the methodologies we use both enable and help define our research. Furthermore, the methodologies we declare in articles permit others to judge the merits of the research we have carried out and to replicate our experiments. As experimental complexity has increased, however, the choice of appropriate methodologies has become an increasingly difficult task [1,2], especially in fields that rely heavily on computational analysis. Indeed there is now a bewildering array of software tools (e.g., [3]) that often perform similar tasks using different methods, making it difficult for individual researchers to keep track of new and existing software, let alone the most suitable software in a specific research area.

Best practice is the most efficient (and effective) declaration of the process that describes the implementation of a specific methodology. Although all of the elements of best practice are routinely considered by researchers, its explicit declaration in biological research is rarely performed. Recording of best practice is an important element of many disciplines such as clinical medicine, e.g., evidence-based medicine [4] and NICE guidelines [5]; medical research, e.g., MRC guidelines [6] and meta-analyses [7]; and in business, e.g., best practice templates [8] and best practice benchmarking [9]. The commonality between these groups is evidence; in order to justify a decision you need evidence upon which to base it. However, most of the current usage of best practice in biological research employs evidence in the context of results with limited regards to the design of experimental protocols [10].

To enable researchers to choose appropriate methodologies, we propose that systems to automatically suggest experimental design templates based on literature-based validation of best practice information will: (i) simplify the design process and (ii) provide a sound scientific basis for the choice of the specific details of an experiment. In order to survey practice in a community and identify best practice we must first be able to collect practice in general. From this data we can then attempt to identify elements therein that may be considered for inclusion into a best practice proposal. Experimental best practice is dependent on its contextual environment. This could constitute the size of the data set to analyse, the field in which the researcher works or the kind of research questions that the research aims to address. We aim to capture elements of context in relation to practice and to incorporate this into our best practice information. Specifically we examine the impact of field allegiance, co-authorship patterns and the overall popularity of methods on experimental practice.

We refer to the complete set of all different practices used by all members of a research community as "community practice". Additionally, we define "best practice" as a subset of community practice, incorporating those elements most scientifically credible and providing the most appropriate choice for any practitioner from the field. Note, when defining best practice we are not necessarily seeking superlatives, but rather a combination of optimal and agreed solutions.

Our implementation of methodology capture involves the application of information extraction techniques to full-text journal literature. Text analysis, text mining and data mining are becoming increasingly popular techniques for information conglomeration. They are suited to the large information resources that are currently available, for example, literature [11,12] or gene expression [13,14] databases. These techniques, for example, have been used extensively in the identification of protein interactions [15,16]. When we combine text mining with the increasing availability of full-text journal articles [17-22], we find it facilitates the automatic identification, extraction and dissemination of experimental methods.

To assess the utility of our approach we selected the field of molecular phylogenetics to act as a test case. Phylogenetics was selected because: (i) the methods used are mainly computational and are implemented by a large but well defined group of software programs, the names of which can be easily collected. (ii) There is significant variety in the methods that different researchers use. (iii) Phylogenetic methods are employed by many scientifically distinct fields of research. (iv) There is debate over which methods should be employed. (v) There is no standard way to communicate or declare the methods and software used in a phylogenetic analysis.

**Figure 1:**
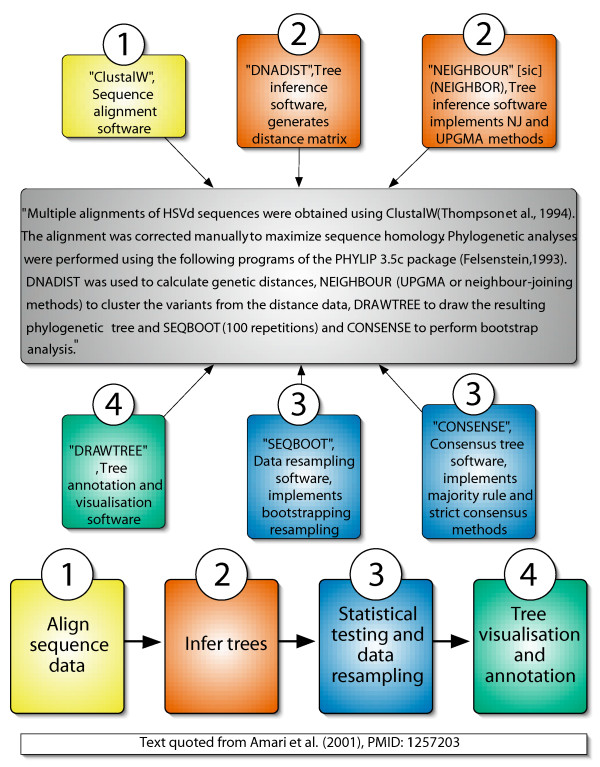
**Model of archetypal phylogenetic experiment**. A model of the archetypal phylogenetic experiment with an example representation of a protocol in text form. Protocol elements are coloured according to their stage (1 to 4) in the model.

The single largest source of phylogenetic and indeed scientific practice is journal literature. Because of the adherence to the scientific method and therefore the need to declare the methods used, each article describing original research should contain text relating to the methods employed. Our approach makes use of this practice resource by operating on the full-text of journal articles. We then search this text for terms that are significant in the description of phylogenetic experiments (see Figure 1 for example).

The set of important methodological terms found in any one article can be said to be a description of the protocol employed in that piece of research. We divide the methodological terms, found in the text, between four key stages: (i) sequence alignment, (ii) tree inference, (iii) statistical testing and data resampling, and (iv) tree visualisation and annotation (Figure 1). The individual protocols are, thus, a model of a scientific experiment that is inferred from the text of the methods described in an article. The phylogenetic terms found in the methods are inferred to describe a task or part of a task and the collection of these tasks is what we term the protocol. Note, our analytical protocol model (Figure 1) is only part of a fully declared workflow that also includes the more mundane aspects: data retrieval, reformatting etc., which may then be transformed into a computer-enabled workflow that can be implemented by a system such as Taverna [23] or Kepler [24]. These are the stages that need to be included for full automation [25,26].

Our approach successfully retrieves a large number of phylogenetic protocols from the scientific literature. Analysing this data, we find that phylogenetic practice over the last 10 years has varied both temporally and between different groups of practitioners. Distinct fields of phylogenetic practitioners can be identified that, although they overlap, are significantly divergent in the protocols they use. We have also identified, using a collaboration network, highly published and widely collaborative researchers for each field. These "expert" researchers design their experiments in very similar ways to the other members of their field and therefore act as useful practice indicators for their field. Our recommendation for producing a best practice proposal for phylogenetics involves a combination of expert practice from each of the most significant fields (for example, evolutionary biology) and the most sophisticated or appropriate practice from all fields.

## Results

A PubMed [27] search for "phylogen*" in titles or abstracts identified 27,259 results, which yielded 24,494 different articles in PDF format. This difference is attributable to incorrect PubMed "link out" data and software difficulties with finding the PDF version of the article from the original link. After processing the 24,494 PDF files, 21,484 articles in plain text remained. Reasons for this difference include a number of PDF files being encrypted, while others contained only scanned images of text.

The result of the community practice gathering and extraction process was 861 unique phylogenetic protocols found in 17,732 different articles. The oldest available article to contain any terms in our data set was published in 1980 and 90% of all analysed articles were published after 1996; before 1996 protocols were retrieved in fewer than 300 articles per year. Thus, we focussed our analysis on the 847 unique protocols identified from 1996 to 2005. We found there are several very popular protocols with most articles (62%) using one of the top 10 most utilised protocols (Additional file 1). This does, however, leave another 851 protocols that have on average only been used seven times each. The 10 most popular protocols all include at least one reference to either neighbor-joining, maximum-likelihood, parsimony or the unweighted pair-group method with arithmetic mean (UPGMA) as a method for phylogenetic tree inference. When assessing the accuracy of protocol identification by comparing our approach to manually annotated text (see methods) we found very high levels of protocol retrieval: precision 89.8%, recall 85.7% and f-measure (f-score) 87.7%.

Now that we have a sample of community practice we can address how practice varies with respect to contextual properties. To do this we investigated the importance of field allegiance and scientific authority (as inferred by co-authorship patterns) in relation to community and best practice. Our first step, therefore, in identifying best practice is to define the fields for which it must be able to cater and whether practice varies between them.

847 different journals are represented in the 21,484 articles that we collected. Out of these, 723 journals are represented in the set of 17,732 articles from which a protocol has been extracted. The 10 most commonly represented journals have published almost 40% of all the articles in our data set. Excluding *PNAS *as a general interest journal, there are three defined journal groups relating to fields of research within these 10 journals: evolutionary biology, microbiology/bacteriology and virology. In addition to the 10 most common journals, we have also classified all journals represented in our article set (see methods) into these journal groups. We found that 17% (3,712 articles) of articles were published in evolutionary biology journals, 22% (4,625 articles) were published in microbiology or bacteriology journals and 11% (2,274 articles) were from journals related to virology. The remaining 50% (10,873 articles) were published in a wide variety of fields.

**Figure 2:**
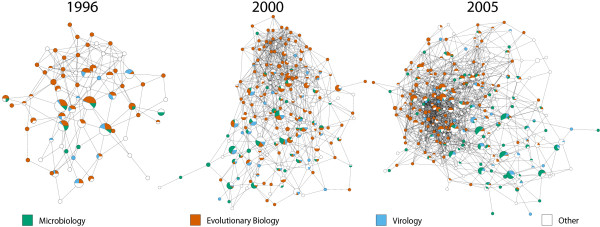
**Usage of protocols by field and through time**. Protocol networks for the years 1996, 2000 and 2005. Nodes represent individual protocols and are sized according to the number of times they were used. Each node is also a pie chart describing the proportion of all uses of that protocol by each field group. Edges denote an F-measure value of greater than 0.75 phylogenetic term similarity between the protocols. The networks shown are the largest connected component after the F-measure threshold was applied.

**Figure 3:**
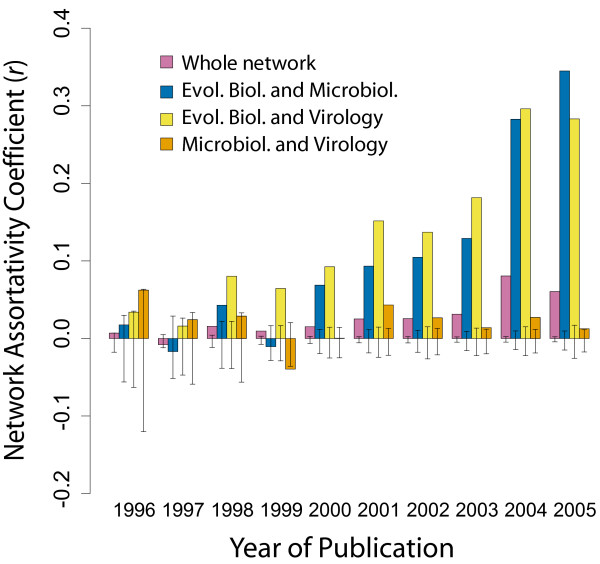
**Field-field network assortativity calculations data**. Bar chart showing the changes in whole network and field-field network assortativity coefficient calculations (*r*). Error bars show 95% confidence interval of distribution of *r *values calculated from 1000 simulated networks (see Methods).

Given that a best practice proposal should be able to cater for all users of phylogenetics, we assessed the differences/similarities between these fields and how they have developed through time. Furthermore, we can use this to assess whether there is methodological communication between fields. To do this we calculate the proportion of articles from each journal group that contained each of the protocols implemented in each year and generate a series of networks (Figure 2) that map the methodological choices made by authors from three different fields during the last 10 years (Additional file 1). These networks indicate that, while there is overlap, a significant shift in methodological preference has occurred between fields. We have used calculations of the network assortativity coefficient (*r*) [28] to highlight changes in methodological choice. In this case a larger *r*-value indicates field-specific method choice. Overall network assortativity and some field-field assortativity comparisons, specifically, Evolutionary Biology/Microbiology and Evolutionary Biology/Virology, have increased throughout this period (Figure 3). Compared to random networks, there is a significantly different increase in overall network assortativity (Figure 3). This is presumably due to the larger increase in assortativity between the Evolutionary Biology field and the other two fields (Figure 3). There was no change in assortativity between the Microbiology and Virology fields, with the values being inside the 95% confidence interval from 1996–1998 and 2000, and when outside the 95% confidence interval only varying between -0.04 and 0.04.

To analyse the pattern of divergence between evolutionary biology and the other two fields, we analysed the usage of terms relating to Bayesian phylogenetic analysis (a relatively new method). Over 60% of evolutionary biology articles published in 2005 included one or more references to a term describing Bayesian phylogenetic analysis of some kind, this compares to 5% of microbiology and 11% of virology articles. This demonstrates that the kind of protocols implemented by the three fields have diverged during the period 1996–2005 and that, in particular, protocols published in evolutionary biology journals have become more distinct from those in the other two journal groups during the same period.

**Figure 4:**
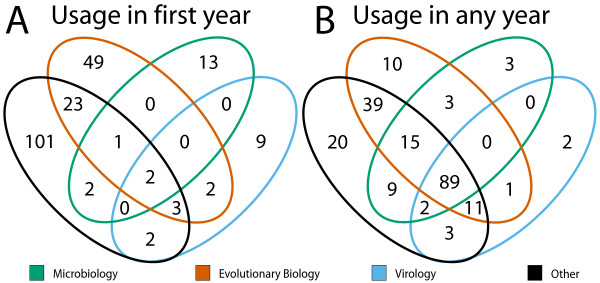
Usage of phylogenetic terms according to field. Venn diagrams showing the usage of phylogenetic terms in articles from all three fields and those from outside the fields. (A) Shows in which field or fields a term was used during the year when it was first mentioned in our corpus; this demonstrates the origin of the term. (B) Shows usage of terms in the three fields (or outside the three fields) but measures usage across all years; this measures communication of the term between fields.

To further investigate the heterogeneous use of different phylogenetic software and methods between fields for each of our phylogenetic terms we analysed: (i) the field or fields in which it was used in its first year (Figure 4A) and (ii) whether it was ever used in each of the fields (Figure 4B). Interestingly, there are a large number of terms that are only used in their first year of reporting (Figure 4A) in evolutionary biology (49/207) and outside of the three fields (101/207). Very few were used in all fields in their first year (2/207), while many more terms (89/207) are used by all fields at some point, and some are only ever used by one (35/207) or two (55/207) of the fields (Figure 4B). Many terms are first published outside the three fields with 80% (81/101) used at least once by one or more of the three fields studied.

It is commonly accepted that much scientific practice is influenced by authority of some kind, be it by role, citations or experience. Therefore authority can be seen as an indicator of best practice. Authorities are, however, almost always specific to a field, for example, a virologist will tend to read virology literature more often than microbiology literature. Given that field-specific research communities exist, we can make an inference on what might be considered "best", or perhaps more accurately commonly published and scrutinised practice, by capturing what is done by the experts in each field. We define our experts as those who are most widely collaborative and also who contribute the most research of publication quality to the community.

To explore expert practice we constructed a collaboration network [29] from our articles and overlaid collaboration metric data. We also labelled our authors according to the journal group in which they most frequently publish. We included only those authors that came from articles from which a protocol has been extracted. This resulted in 45,290 unique authors and 190,530 collaborations between them. Each author was represented as a node and each collaboration as an edge. We assigned two sets of attributes to the edges: these were the number of collaborations (edge weights 1) between the two authors connected by the edge and the number of collaborations divided by the number of authors on each article (edge weights 2) of which they were co-authors [30]. This weights collaboration between authors on articles with a small number of co-authors more highly than articles with many co-authors.

**Figure 5:**
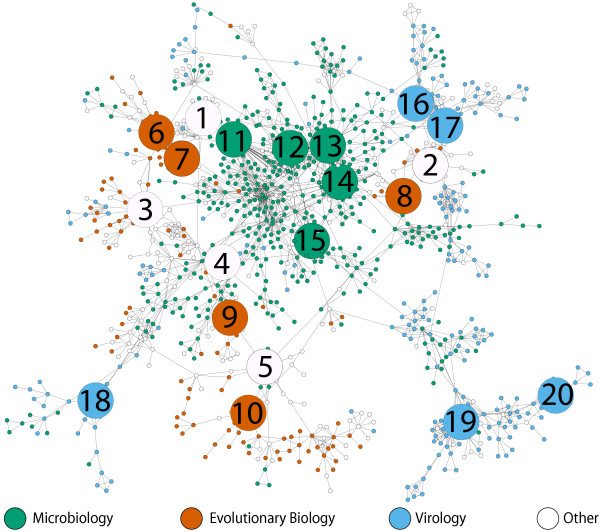
**Co-authorship network highlighting most expert authors**. Co-authorship network according to research field. Nodes represent individual authors, edges represent three or more co-authorships between the two connected authors. The 20 expert authors (see Methods) are represented by larger nodes with numbered labels. Author Names, 1: Koonin, E.V., 2: Pace, N.R., 3: Wang, Y., 4: Zhang, Y., 5: Doolittle, W.F., 6: Hasegawa, M., 7: Okada, N., 8: Nei, M., 9: Roger, A.J., 10: Meyer, A., 11: Falsen, E., 12: Collins, M.D., 13: Stackebrandt, E., 14: Schumann, P., 15: Yoon, J.H., 16: Orito, E., 17: Mizokami, M., 18: Webster, R.G., 19: Sharp, P.M., 20: Gessain, A.

In order to identify authors who were most active in this network, we restricted the node set to include only those that had co-authored three or more articles with one or more other authors. This reduced the largest connected component of the resultant network to 1,112 nodes and 2,412 edges; we refer to this network as the reduced collaboration network (Figure 5). When we consider the reduced collaboration network of authors (Figure 5) who have published in our phylogenetics corpus, we see a field-specific pattern similar to that in Figure 2. There are many authors who tend not to collaborate regularly with others outside their field (visible as the clusters of nodes of a single colour) and then there are other authors who link the clusters through interdisciplinary collaborations.

We quantified the field specific structure in Figure 5 in the same way as Figure 2 (see methods). Overall network assortativity is 0.36 with fields being almost equally assortative with respect to each other (Evolutionary Biology/Microbiology: 1.0, Evolutionary Biology/Virology: 0.977 and Microbiology/Virology: 0.959). Note, the assortativity value of 1.0 between evolutionary biology and microbiology indicates there are no co-authorships between authors from these fields in this reduced network. Using a metric of collaborative activity (see methods) we have highlighted the five most active researchers from each of the three fields as well as five from outside the fields. Interestingly, the protocols employed by these 20 most highly active authors in the network are very similar to those used by the rest of the community. The 10 most frequently used protocols by the non-experts (used 73% of the time) are used almost as frequently by the experts (74%). The experts have co-authored 1,001 articles between them and these articles have made use of 26 protocols unique to their group.

## Discussion

Our survey of phylogenetic practice over the last 10 years has found a large range of experimental protocols declared at varying levels of detail. This has created an environment of both consensus and variation. We have authors reporting methods that are commonly used by hundreds of authors, and others who create highly bespoke experimental protocols of their own design and remain the sole practitioners of these protocols. Context is clearly important. It could be that these highly specific protocols are used to answer highly specific research questions. Until we are able to reliably capture detailed contextual information of this kind we will not know whether this is the case. In addition our results highlight the need for better recording and communication of experimental methods.

The observed field-specific and temporal variations (Figures 2 and 3) in community practice suggest that an opinion of what constitutes best practice is changing through time and depends to a large extent on the field to which an individual belongs. In particular, we find there has been a significant shift in the methodologies used by the evolutionary biology field and the microbiology and virology fields, which is partly attributable to the differential use of Bayesian inference. We have analysed the emergence and spread of different phylogenetic terms in the three fields (Figure 4), and find a large number of terms unique to the evolutionary biology field (in their first year of usage, Figure 4A) and that a large number of terms are never used outside of this field (Figure 4B), except for when they are used in ungrouped journal articles. The number of terms being first used outside the three fields (101/207, Figure 4B) is presumably a reflection of the specialised nature of phylogenetic methods and software. The authors who develop new methods and software tend not to publish in the same kind of journals as those who use their innovations.

Almost all of the 10 protocols used most commonly by the phylogenetics community represent a valid choice (except those using UPGMA (see [31,32])) for a researcher new to the field. Common community practice is therefore a good starting point upon which to build a best practice proposal. It does, however, lack those features unique to specific fields and the requirements of specific users.

Our analysis suggests that communication of methods is not only difficult, i.e., researchers apparently use many different computer programs for generally the same type of analysis, but it is also hampered by inefficient information exchange between practitioners of phylogenetics. The latter appears to be due to academic specialism, in that researchers tend to first look to others from their own field when choosing methods. Interestingly, expert authors tend not to use protocols that are distinct from others in their field. This makes the protocols used by experts in each field a valuable indicator of the kind of protocols commonly used in their respective fields and, thus, a useful short-cut to identifying best practice for that specific field.

Our model-based method for protocol extraction (Figure 1) has permitted the construction of representations of protocols that have a direct link with the physical implementation of that protocol. The use of full-text journal articles was necessary due to the information we were endeavouring to capture, i.e., experimental methods. Many other text mining projects would be significantly enhanced with the use of full-text. An abstract is only an abbreviated summary that presents the most important findings from a piece of research and very briefly places them in context. Its function is to act as a point of entry to a complete manuscript. Much potentially important information, and nuance, will only be found in the full manuscript. Thus, given the number of different scientifically interesting elements [17-19,22] contained in a full article, that are usually not present in the abstract, text mining research needs to make more use of full-text.

The model-based method for protocol extraction also allows us to organise method terms according to the order in which they would have been used in the experiment rather than their order in the text. This is a powerful element of our approach that could lead to further work on automated suggestion of methods or software for a given task or part of a protocol. The model can also allow us to account for missing information in our extracted protocols, so that if an extracted protocol does not contain any terms related to a single part of the model (Figure 1), we can still analyse the other parts of the protocol for which we do have information. This is an important feature for analysing information automatically derived from text, which is often sparse, with some elements well described and easy to identify and analyse, and others described indirectly via citation, figure legends or supplementary information. Our structured approach to capturing protocols from full-text articles could be applied to any discipline of science where the methods used can be broken down into individual sequential stages. For example, a simple molecular biology task to sequence a genic region from a fruit fly could be broken down into: DNA extraction, purification, amplification, sequencing and chromatogram analysis. As with a phylogenetic protocol several terms could map on to each one of these stages, for example, PCR or bacterial cloning could be used in the amplification step.

In the increasingly specialised world of scientific research, our results demonstrate the need for strong collaboration and communication between fields of research, especially between those implementing similar experimental designs. Best practice information derived from whole disciplines rather than small research communities allows us to share information between a larger number of researchers who may have no knowledge of new innovations in other fields. Best practice also supports replication of results and standardisation of practices by providing protocols that can be reused in many different research areas and which produce comparable results. Comparable results are of particular importance in phylogenetics at the moment, with the advent of phylogenomics [33,34] and projects attempting to construct and represent the full tree of life [35-37].

## Conclusion

Our capture of protocols from a large group of researchers has allowed us to reliably survey the current state of practice in the design of protocols in the field of molecular phylogenetics. This information is useful for monitoring best practice versus new trends and directions in the community as well as identifying from where they originate.

The capture of best practice is a non-trivial task; in this case we have found that the practice of highly published authors acts as a good proxy for that of others in the same field. We have also defined how the main fields have altered methodologically over time. The evolutionary biology field, in particular, has diverged from the others and these changes are characterised by the use of new and more advanced methods. This suggests that the practice of expert authors in evolutionary biology (Table 1) is the closest to contemporary best practice for phylogenetic experimental design. Notwithstanding data specific issues, a protocol combining those methodological elements present in the protocols of the evolutionary biology group combined with elements from protocols of the experts in the specific field offers an appropriate choice for any researcher. We also envisage the tailoring of best practice to individual users needs. This would determine the user's experimental context, including such information as data set size, any potential idiosyncrasies of the data, the level of analytical detail required, possible time constraints etc. Such information could be used to modify a base protocol to the data and users needs.

**Table 1:** Expert evolutionary biology methodological protocols.

**Protocol**	**Usage (number of articles)**
Neighbor-joining, Parsimony, Maximum-likelihood	15
Neighbor-joining, Parsimony, Maximum-likelihood, JTT model	9
Maximum-likelihood	8
Neighbor-joining	8
Neighbor-joining, Parsimony, Maximum-likelihood, HKY model	8
Neighbor-joining, Maximum-likelihood	6
Maximum-likelihood, Bayesian	5
Parsimony, Maximum-likelihood	5
Neighbor-joining, Maximum-likelihood, HKY model	5
Neighbor-joining, Parsimony, Maximum-likelihood, Bayesian	5

A sample of the most commonly implemented methodological protocols used by the top five expert authors from the evolutionary biology field

Currently practice capture requires extensive effort to identify community-wide information from a field where the choices of methods are well defined and described. We propose that an explicit model of an experimental protocol represented as a workflow [26] will improve communication, sharing and ranking of experimental protocols and will support one of the central tenets of the scientific method, that of replicable results [1,2,38,39]. Importantly, this computer-enabled workflow should contain all parameters and methods of all elements of the experiment, with stable connections to implementations of these methods that are accessible to all. Specific phylogenetic protocols (represented as implementable workflows) could then be associated with quality metrics. For example, quantification of usage based on the number of published articles using the protocol, protocols associated with specific authors and types of data, specialist protocols that integrate additional methodologies, e.g., the detection of recombinant sequences. Work such as this will benefit from concerted effort on the subject of context capture and how to capture the real aims of a study. This will complete the linking of methods with aims permitting researchers to efficiently tailor experimental solutions to specific research projects.

## Methods

### Article identification

We collected a set of journal articles in PDF format identified by a PubMed search defined as "phylogen* [Title/Abstract] AND (full-text [sb]) AND ("X" [PDat]:"Y" [PDat])" where X and Y are dates used to restrict the number of results returned. The search was performed 01/04/06. The Quosa Information Manager [40] was used to download the PDF files.

### PDF file text extraction

Text was extracted using the pdftotext executable from the xpdf package [41] provided by foolabs. The executable was run with default settings.

### Methodological term identification

We used manually tested and designed regular expression patterns to identify the methods declared in the text. A manually created controlled vocabulary of 258 important names and terms (Additional file 2) was used to store and group these patterns. The controlled vocabulary is an XML document that groups terms according to their methodological nature. The software names were taken from Professor Joseph Felsenstein's page of phylogenetic programs [3]. Other terms were manually created using the phylogenetics primary literature.

### Protocol Formation

We constructed two forms of the protocol from each article. The first included the term matches for all terms in the vocabulary. The second form only includes terms that are classified as a type of "phylo_method" or "phylo_model" in the vocabulary document.

### ISI Journal Citation Report categorisation

Classification of journals is determined by the ISI Journal Citation Report (JCR) service, which classifies journals by discipline. The three journal subject categories used are "Evolutionary Biology", "Microbiology" and "Virology" with 16, 51 and 15 of the journals in our data set being present in these categories respectively. All journals not present in these groups were labelled "Ungrouped".

### Protocol similarity

We use the F-measure [42] to determine similarity between protocols. The F-measure value gives an indication of the number of shared terms between two protocols as a fraction of all the terms found in the two protocols. This gives a measure of methodological similarity whilst also normalising for the number of terms found. The F-measure is calculated between all pairs of protocols. The F-measure is calculated as follows.

$$\text{F}-\text{measure}=\frac{2c}{n_{1}+n_{2}},$$

where

*c *= the number of terms common between the two protocols,

*n*_1 _= the total number of terms in the first protocol, and

*n*_2 _= the total number of terms in the second protocol.

### Author names

We used the surname and all initials method of author name construction [43]. This can lead to multiple author names that may refer to the same author. However, we felt that this would not bias the structure of the network significantly given that most authors tend to co-author most of their articles with a similar group of collaborators. This method can also avoid the problem of common surname/first initial combinations referring to multiple authors.

### Protocol networks

The protocols in the protocol networks in Figure 2 are present in the largest connected component from a network of all unique protocols found in this study that have an F-measure similarity score of greater than 0.75 and were captured from an article published in the given year. The protocols are in this case constructed from the terms found in the article that are classified as part of the "phylo_method" or "phylo_model" sections of our term vocabulary. This eliminates unimportant software implementation detail from the protocols and allows us to analyse the specific methodological choices made in each article.

We generated the pie node networks in Figure 2 using the GenePro [44] plugin for Cytoscape [45]. Each pie node gives a visual representation of the proportional use of each protocol by each journal category.

### Author field/journal group affiliation

Each author node present in the reduced collaboration network was assigned a journal group label. These were the same set of labels used in the protocol networks (Figure 2). If the author had published more than half (the majority) of their articles in one of the journal groups then they were labelled with that group. Otherwise they were labelled as "ungrouped" and appear as white nodes in the network.

### Expert identification

For each node in the reduced collaboration network, we calculated the sum of the values of edge weight two for every connected edge. We then used five nodes with the highest value of this metric from each of the three fields and from the ungrouped authors as our set of 20 experts.

### Term identification error analysis

To test the accuracy of our term matching we manually annotated the methods section or section of text most descriptive of methodological detail for 50 randomly chosen articles from our corpus. We annotated all pieces of text that referred to any of the phylogenetic entities that are present in the controlled vocabulary.

### Network assortativity calculations

**Figure 6:**
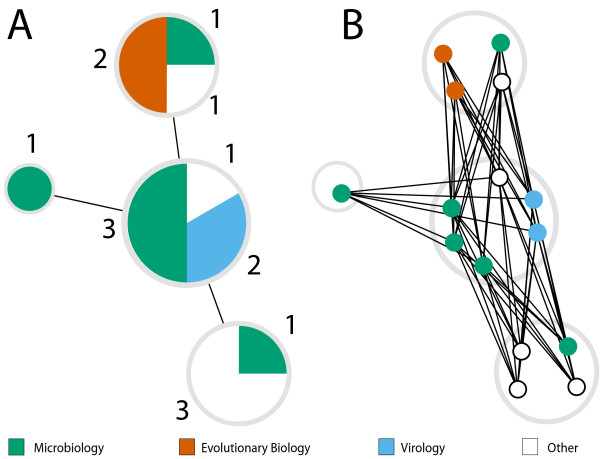
**Example of discrete network construction**. Example of discrete network construction. Numbers denote the number of articles from each group in the pie node. (A) The starting pie node network. (B) The resultant discrete network model used for assortativity calculations.

The calculation of the network assortativity coefficient (*r*) requires that each node is given a class label [28,46]. Because our nodes are composite structures that describe the number of articles from each field using the particular protocol, we calculated *r *using a discrete model of our network. Each pie node becomes a set of nodes of size *n *where *n *equals the number of articles that used the protocol. These nodes are then assigned class labels based on the field composition of each pie node. We then create edges between all new nodes that were part of a pie node pair (Figure 6).

### Network simulations and error

For each of the years between 1996 and 2005 (inclusive) we calculated the *r *value, the coefficient of network assortativity on the whole network and for all pairwise field comparison subnetworks. To gain a measure on the error of these values we simulated 1,000 randomised networks for each year and performed the same network assortativity calculations on these networks. The randomisation process maintained everything from the original network apart from the class labels assigned to each node. These labels were shuffled and reassigned to each node. We then calculated the 95% confidence interval of the distribution of simulation results and these values are presented in Figure 3 as error bars.

### Author metadata

Author metadata for each article was extracted from the *eSummary *NCBI eUtils service [47]. We used the PubMed ID (PMID) as a unique identifier for each article. The PMID for each article was obtained by Quosa when the article was originally downloaded.

### Network visualisation

The networks in Figure 2 were visualised using Cytoscape version 2.3.2 [45] with yFiles organic layout and the GenePro plugin. The network in Figure 5 was visualised using Cytoscape version 2.6.0 with force-directed layout and using default settings.

## Authors' contributions

JME, RDS and DLR designed the work. JME carried out the text mining, network construction, error analysis and drafted the manuscript. JWP participated in the design of the simulation and statistical analysis of networks. All authors edited and approved the final manuscript.
